# Racial/Ethnic Differences in the Modifying Effect of Community Violence on the Association between Paternity Status and Preterm Birth

**DOI:** 10.1155/2017/3479421

**Published:** 2017-11-27

**Authors:** Timothy O. Ihongbe, Saba W. Masho

**Affiliations:** ^1^Division of Epidemiology, Department of Family Medicine and Population Health, School of Medicine, Virginia Commonwealth University, Richmond, VA, USA; ^2^Department of Obstetrics and Gynecology, School of Medicine, Virginia Commonwealth University, Richmond, VA, USA; ^3^Institute for Women's Health, Virginia Commonwealth University, Richmond, VA, USA

## Abstract

Preterm birth (PTB) is a major public health concern in the US. Lack of established paternity has been linked with increased risk of PTB. Community violence (CV) may modify the association, and racial/ethnic differences may exist. Using a geographically defined cohort of women in Richmond, Virginia (*N* = 27,518), we examined racial/ethnic differences in the modifying effect of CV on the association between paternity status and PTB. Results showed that lack of established paternity was associated with incremental greater odds of PTB across CV quartiles in NH-Whites (quartile-1: AOR = 1.42, 95% CI = 0.95–2.12; quartile-2: AOR = 1.45, 95% CI = 0.57–3.71; quartile-3: AOR = 3.12, 95% CI = 2.67–6.32), NH-Blacks (quartile-1: AOR = 1.16, 95% CI = 0.85–1.58; quartile-2: AOR = 1.32, 95% CI = 0.82–2.12; quartile-3: AOR = 1.64, 95% CI = 1.24–2.16), and Hispanics (quartile-1: AOR = 1.29, 95% CI = 0.65–2.55; quartile-2: AOR = 1.34, 95% CI = 0.67–2.69). Odds of PTB were highest among NH-White women. Public health practitioners should be aware of the negative effect of lack of paternal presence on PTB in women resident in high violence rate communities and racial/ethnic differences that exist.

## 1. Introduction

Preterm birth, defined as the birth of a baby before 37 completed weeks of gestation, is an important cause of perinatal mortality in the US and contributes significantly to perinatal and infant morbidity [1]. About 1 in every 10 infants born in the US is born preterm [2]. Complications of preterm birth are the leading cause of death among children under 5 years of age and are responsible for nearly 1 million annual deaths globally [3]. Additionally, infants born preterm have higher rates of both short- and long-term health complications and lifelong disabilities which include mental retardation, learning and behavioral problems, cerebral palsy, lung problems, vision and hearing loss, diabetes, high blood pressure, and heart disease [4–6]. Furthermore, children who are born preterm have a higher risk of increasing difficulties with complex language functions compared with term-born children [7].

For decades, research has focused on identifying maternal risk factors linked to the delivery of a preterm baby. Some of the known risk factors include previous preterm delivery, multiple gestation, underweight and obesity, short and long interpregnancy intervals, tobacco use, bacterial vaginosis, and low socioeconomic status, among others [8, 9]. However, these maternal risk factors have only been able to explain about 25% to 30% of preterm births in developed countries [10]. Recently, paternal factors have been linked with an increased risk of preterm birth. Such paternal factors include paternal depression, father's attitude towards the pregnancy, father's health behaviors during the prenatal period, relationship between father and mother, and paternity status [11–14]. Alio et al., in a study to examine the impact of paternity status on fetoinfant morbidity in women in Florida, reported that women with absent fathers across all racial-ethnic groups had higher risks of preterm birth than their counterparts with involved fathers [11]. Similarly, Masho et al., utilizing Virginia birth registry data, examined the population of women in Virginia with singleton live births who reported paternal acknowledgement. They reported that women with no father acknowledgement were more likely to have preterm babies [12].

One mechanism underlying the association between paternity status and preterm birth is maternal stress. Maternal stress is believed to play a major role in increasing the risk of preterm birth by activating the neuroendocrine system which triggers the release of mediators such as adrenalin, corticotrophin-releasing hormone, cortisol, and other chemical messengers that increase the risk of preterm birth [15, 16]. The presence of a father during pregnancy has been suggested to help to reduce maternal stress by providing emotional, logistic, and financial support [17]. Furthermore, the presence of a father during pregnancy may help to promote healthy maternal behaviors which may reduce the risk of preterm birth [18].

*The Role of Community Violence and Race/Ethnicity*. Community violence has been reported to be associated with preterm birth [5, 19–21]. Foureaux Koppensteiner and Manacorda using microdata from the Brazilian vital statistics on births examined the impact of in utero exposure to community violence (measured by homicide rates) on preterm birth [19]. They reported that exposure to community violence during the first trimester of pregnancy led to an increase in the risk of preterm birth. Further, Messer et al., examining maternal violent crime exposure in the city of Raleigh, North Carolina, USA, reported that violent crime characterized as a community attribute was positively associated with preterm birth [5]. Given that community violence has been postulated to increase the risk of preterm birth through maternal stress [22, 23], it is plausible that maternal exposure to community violence in women with absent fathers or no established paternity may further increase the risk of preterm birth, such that as the rate of community violence increases, the risk of preterm birth may also increase. Furthermore, racial/ethnic differences have been reported in maternal exposure to community violence. For example, Messer et al. in a study to examine violent crime exposure and adverse birth outcomes among a geographically defined cohort of women in Raleigh, North Carolina, USA, reported that about 62% of non-Hispanic (NH) White women resided in census blocks that had low rates of community violence as compared to 21% for NH Black women [5]. Conversely, almost half of NH Black women (47%) resided in census blocks that had high rates of community violence, as compared to 9% for NH White women. This therefore suggests that the modifying effect of exposure to community violence on the association between paternity status and preterm birth may vary across race/ethnicity.

Despite the substantial body of literature that have examined the effect of paternity status on poor birth outcomes such as preterm birth [11, 12, 17, 24], the modifying effect of community violence on the risk of preterm birth in women with no established paternity or paternal presence has not been studied. Improving the understanding of how community violence modifies the association between paternity status and preterm birth is important to effectively address preterm birth in the population of women with absent fathers. By understanding how community violence modifies the association between paternity status and preterm birth and how the modifying effect varies across race/ethnicity, research can point to ways in which community violence, paternal absence in pregnancy, and preterm birth can be addressed across different racial/ethnic groups in the population. The aim of this study, therefore, is to examine the modifying effect of community violence on the association between paternity status and preterm birth in a geographically defined cohort of women in Richmond, Virginia, and to determine if the modifying effect differs across maternal racial/ethnic groups. We hypothesize that (1) lack of established paternity will increase the risk of preterm birth, and the risk will be greater with increasing levels of community violence; (2) the modifying effect of community violence will vary across racial/ethnic groups, with NH Hispanic Black women experiencing a greater impact relative to NH White and Hispanic women.

## 2. Materials and Methods

### 2.1. Data Source and Study Population

Live birth records, police crime reports, and census data for Richmond, Virginia, were utilized in this study to create a geographically defined cohort of women. Birth records of all singleton live births were obtained from the Virginia Department of Health Vital Statistics for a 10-year consecutive period (2004–2013). Birth records contained information on maternal sociodemographic history (e.g., age, race, and education), reproductive history (e.g., gestational age at delivery and prenatal care attendance), and risky behaviors (e.g., tobacco and alcohol use). In addition, live birth records included information on presence or absence of the father's first and/or last name. Live birth records were geocoded using maternal addresses using ArcGIS version 10 (ESRI 2011. ArcGIS Desktop: Release 10.1. Redlands, CA: Environmental Systems Research Institute) to identify residential census tracts. About 89 percent of the birth records were successfully geocoded. The live birth records were then linked to the 2010 US census data using the unique residential census tract identifiers described above. Crime reports were obtained from the Richmond Police Department, Virginia. The police crime report provided data on crime incidents which occurred during the 10-year period from 2004 to 2013 in Richmond, Virginia. Crime data included all Class A reportable offenses (aggravated assault, kidnapping, homicide, sexual assault, robbery, theft, burglary, larceny, arson, destruction of property, and vandalism) involving youths aged 10–24 years. The mean incidence rate of violence was calculated for each census tract for the 10-year period and merged with the geocoded live birth record. The final data had a 2-level hierarchical structure with live births nested in 64 census tracts. Detailed description of the data has been reported elsewhere [25]. The study population included women with valid gestational age at delivery who had father's first and/or last name documented on the birth certificate (*N* = 27,518).

### 2.2. Measures

*Outcome*. Preterm birth was defined as the birth of a baby before 37 completed weeks of gestation. Gestational age at birth was categorized into 2 levels: preterm birth (<37 weeks) and term births (≥37 weeks). Gestational age was determined using the clinical estimate computed by the physician. In situations where the clinical estimate of gestational age was missing or implausible (<1%), gestational age was computed by taking the interval between date of last menstrual period reported by the mother at her first prenatal visit and the date of delivery.

*Exposure*. Paternity status was measured as a binary variable (paternity established or paternity not established) using the presence or absence of the father's first and/or last name on the birth certificate, as has been used previously in the literature [11, 24]. The presence of the father's first and/or last name on the birth certificate has been shown to closely mirror paternal presence during the pregnancy period. Teitler, in a study to examine father involvement, child health, and maternal health behavior, reported that the proportion of fathers who were present during the pregnancy of their partner and the proportion of fathers who had their names listed on the birth certificate were very similar (87% and 90%, resp.) [18].

*Effect Modifiers*. Community violence was measured at the census tract level. It was obtained by aggregating Class A reportable offenses (aggravated assault, kidnapping, homicide, sexual assault, robbery, theft, burglary, larceny, arson, destruction of property, and vandalism) involving youths aged between 10 and 24 years to produce a single violence variable. Violence rates were calculated for each census tract and the mean violence rate per 1,000 youth population during the 10-year period was computed. The mean violence rate was then categorized into quartiles, ranging from low (quartile 1) to high (quartile 4).

Maternal race/ethnicity was categorized as NH Whites, NH Blacks, and Hispanics. Other NH racial/ethnic groups such as Asians, American Indians, Hawaiians, and Alaskan Natives were excluded due to small numbers.

*Covariates*. Factors examined include individual- and community-level factors. Individual-level factors include maternal age (<19, 19–24, 25–34, and ≥35 years), maternal education (less than high school, high school graduate, and more than high school), insurance (private, Medicaid, and self-pay), tobacco use (yes or no), alcohol drinking (yes or no), previous preterm birth (yes or no), parity (0, 1, 2, or 3 or more), and adequacy of prenatal care (inadequate/intermediate, adequate, or adequate plus). Prenatal care adequacy was defined using the Kotelchuck index which takes into account when prenatal care was initiated and how many visits were completed, controlling for estimated gestational age at delivery [26]. Community-level factors included percentage poverty level (percent of residents living below 100% of the federal poverty level), percent of female-headed households, and percentage of Black population.

### 2.3. Statistical Analysis

**Figure 1:**
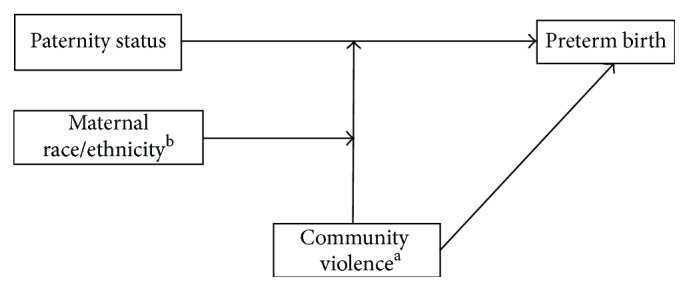
Conceptual model for 3-way interaction effect of community violence and race/ethnicity on the association between paternity status and preterm birth. ^a^Modifying effect of community violence on the association between paternity status and preterm birth. ^b^Modifying effect of maternal race/ethnicity on the modifying effect of community violence.

All analyses were conducted using SAS 9.4 (SAS Institute, Cary, NC). Descriptive analyses were conducted to determine the characteristics of the study population across individual- and community-levels. The GLIMMIX procedure in SAS was then used to fit four multilevel logistic regression models for the response variable (preterm birth) using a binary distribution and logit link function. Model 0 was an unconditional model (i.e., a model containing no predictors). This will facilitate the calculation of the intraclass correlation coefficient (ICC) to estimate how much variability in the response variable is explained by the individual and community-level factors. Model 1 (unadjusted model) examined the association between paternity status and preterm birth. Model 2 was fit to control for individual-level factors. Model 3 controlled for individual-level factors from model 2 in addition to community-level factors. Model fit was assessed using the deviance test utilizing Laplace estimation and multiple testing correction was done using the Bonferroni correction (0.05/4 = 0.0125). To explore racial/ethnic differences in the modifying effect of community violence on the association between paternity status and preterm birth, community violence and race/ethnicity were entered as a three-way interaction term (paternity status *∗* community violence *∗* race/ethnicity) to the unadjusted model and statistically tested using the likelihood ratio test (*p* = 0.0134). As recommended in the literature [27], all lower order (two-way) interaction terms (paternity status *∗* community violence, paternity status *∗* race/ethnicity, and community violence *∗* race/ethnicity) were also included in the model. The best fitting model was thus stratified across quartiles of community violence and race/ethnicity. The conceptual model is shown in Figure 1. Statistical significance was set a priori at 5%.

## 3. Results

Table 1 shows the characteristics of the study population by paternity status. Approximately 59% of women (*p* < 0.0001) reported no established paternity. A greater proportion of NH Black women, adolescents and young adults, women on Medicaid, high school graduates or less, women with parity of 2 or higher, and women with inadequate prenatal care were without established paternity (*p* < 0.0001). For community-level factors, a greater proportion of women in the upper quartiles of community violence reported no established paternity (<.0001) and on average, a greater proportion of female-headed households, households below 100% of the Federal Poverty Level, and NH Black population reported no established paternity (<.0001).

Table 2 shows deviance tests for four competing models (models 0, 1, 2, and 3) previously described to determine the best fitting model. Using the -2Loglikelihood test and associated degrees of freedom to calculate the chi square statistics for nested models, model 3 (paternity status, individual-level, and community-level characteristics) was determined to have the best fit. The ICC showed that community-level factors explained over 2% of the variability in preterm birth (not shown in table). However, although the variability explained by community-level factors was small, there was a statistically significant (*p* = 0.0297) amount of variability in the log odds of preterm birth between the census tracts, and so community-level factors were retained. This was consistent with the deviance tests.

Multilevel regression analyses of paternity status and preterm birth stratified by community violence and race/ethnicity for the best fitted model (model 3) are shown in Table 3. Compared to women who had births with established paternity, lack of established paternity was associated with incremental greater odds of preterm birth across quartiles of community violence (from quartiles 1 to 3) in NH White, NH Black, and Hispanic women, such that as community violence rates increased, the odds of preterm birth also increased. The odds of preterm birth were highest among NH White women (quartile 1: AOR = 1.42, 95% CI = 0.95–2.12; quartile 2: AOR = 1.45, 95% CI = 0.57–3.71; and quartile 3: AOR = 3.12, 95% CI = 2.67–6.32) and lower for NH Black women (quartile 1: AOR = 1.16, 95% CI = 0.85–1.58; quartile 2: AOR = 1.32, 95% CI = 0.82–2.12; and quartile 3: AOR = 1.64, 95% CI = 1.24–2.16) and Hispanic women (quartile 1: AOR = 1.29, 95% CI = 0.65–2.55 and quartile 2: AOR = 1.34, 95% CI = 0.67–2.69). Regression estimates for the 2 highest quartiles of community violence for Hispanic women could not be ascertained due to small numbers. For quartile 4, the odds of preterm birth in both NH White and NH Black women were lower than the odds observed in quartile 3 (AOR = 2.99, 95% CI = 1.17–7.66 and AOR = 1.05, 95% CI = 0.74–1.49, resp.).

## 4. Discussion

This study shows that lack of established paternity is associated with greater odds of preterm birth in women, and the odds of preterm birth increase with higher rates of community violence for NH White, NH Black, and Hispanic women. These findings support our study hypothesis and are consistent with previous studies that reported greater risk of preterm birth in women with no established paternity [11, 28]. The presence of a father during the pregnancy period has been shown to mitigate the effect of stress on the risk of preterm birth [15]. Paternal presence has been suggested to be one of the most significant sources of support in relation to prenatal stress [29]. The presence of a father during pregnancy may help to reduce maternal stress by providing emotional, logistic, and financial support [17]. Furthermore, the presence of a father during pregnancy may help to promote healthy prenatal behaviors [18]. This was evident in the current study as women who reported no established paternity were more likely to engage in unhealthy prenatal behaviors such as tobacco use and inadequate utilization of prenatal care services.

Furthermore, the odds of preterm birth in women who had births with no established paternity were shown to increase with increasing rates of community violence. This finding was not surprising and is consistent with our study hypothesis, as previous studies have reported that the risk of preterm birth increases with higher rates of community violence [20, 21]. It is possible that community violence may have a synergistic effect on the risk of preterm birth in women who had births with no established paternity. A surprising finding, however, observed in this study was the higher odds of preterm birth seen in NH White women with no established paternity, relative to NH Black women, who were exposed to community violence. This finding was contrary to our study hypothesis. We hypothesized that NH Black women with no established paternity who were exposed to community violence, will have the greatest odds of preterm birth. Previous studies have reported that NH Black women with no established paternity, relative to NH White and Hispanic women, had the greatest odds of preterm birth [11]. However, findings from this study suggest that community violence may have a greater impact on the association between paternity status and preterm birth in NH White women than in NH Black women. The impact of community violence on NH White women may seem to be due to fear or perception of community violence rather than the actual effect of community violence. Evans and Fletcher in a study to test alternative hypotheses on fear of crime reported that White women were more fearful of crime than other racial groups despite a decreased risk of victimization [30]. This hypothesis is further supported by findings from the current study for maternal exposure to community violence. Among women resident in census tracts with the highest rates of community violence (quartile 4), NH White women comprised about 16%, compared to 82% for NH Black women. Conversely, among women resident in census tracts with the lowest rates of community violence (quartile 1), NH White women made up over half (51%) of the population, while NH Black women comprised 36%.

Another important finding observed in this study was the attenuation of the odds of preterm birth in women who had births with no established paternity and were exposed to the highest rates of community violence (quartile 4), in both NH White and NH Black women. This finding was in contrast to previous studies that have shown that women in areas with high rates of community violence have the greatest risk of preterm birth [20, 21]. The attenuation of the association may be due to development of resilience in women who were exposed to very high levels of community violence [31, 32]. When women are continuously exposed to high levels of community violence, they may be forced to develop coping styles and strategies to respond to the constant threats to their safety and well-being [31, 32]. The development of maternal resilience may thus have partly buffered the negative effect of community violence. Unfortunately, data was not available to assess chronicity of exposure to community violence.

### 4.1. Strengths and Limitations

This study utilized a geographically defined cohort of women with singleton live births in Richmond, Virginia, USA, which has one of the highest crime rates in the US [33]; hence, we were able to adequately capture community violence. Additionally, actual rates of community violence were utilized, as opposed to self-reported (subjective) community violence exposure. Lastly, we made use of robust statistical methods (PROC GLIMMIX) which adequately handled hierarchical generalized linear models in the analyses. However, this study was not without limitations. First, the use of paternity status as a proxy for paternal presence may not be the most accurate method to measure paternal presence. Second, due to limitations in the data, we were unable to examine paternal involvement or reasons for lack of established paternity. Third, measurement of community violence was limited to youths between 10 and 24 years, and did not include violence perpetrated by older adults (>24 years). The impact of this limitation may be minimal as adolescents and young adults have been reported to have the highest rates of violence perpetration in the US [34]. Lastly, due to unavailability of certain variables in the data set, we were unable to measure some important variables such as maternal stress, receipt of general social support, and chronicity of exposure to community violence, which may have been helpful to explain the relationship between paternity status and preterm birth.

## 5. Conclusion

This study demonstrated that lack of established paternity is associated with increased odds of preterm birth, and the odds of preterm birth increased as the rate of community violence increased in NH White, NH Black, and Hispanic women. Community violence was also shown to have differentially greater modifying effect on the odds of preterm birth in NH White women who had no established paternity, relative to NH Black and Hispanic women. Healthcare providers and policy makers should be aware of the negative effect of lack of paternal presence on preterm birth in women resident in communities with high rates of violence and the racial/ethnic differences that exist. Intervention programs to provide support to pregnant women with no paternal presence who are resident in communities with high rates of violence are needed. Receipt of social support may help women to better cope with stressors that may arise from lack of paternal presence and exposure to community violence [35].

## Figures and Tables

**Table 1:** Characteristics of study population by paternity status, Richmond, Virginia, 2004–2013.

Characteristics	Total *N* = 27,518 %	Paternity not established *N* = 16,146 %	Paternity established *N* = 11,372 %	Chi square^a^
*Individual-level characteristics*				
All participants	100	58.7	41.3	<.0001
Age				<.0001
<19	7.4	11.8	1.2	
19–24	33.5	47.2	14.0	
25–34	46.5	35.1	62.8	
≥35	12.6	5.9	22.0	
Race/ethnicity				<.0001
Non-Hispanic White	31.3	9.7	62.6	
Non-Hispanic Black	57.6	78.2	27.6	
Hispanic	11.2	12.1	9.9	
Education				<.0001
Less than high school	24.7	35.4	9.7	
High school graduate	27.7	39.4	11.3	
More than high school	47.6	25.3	79.1	
Insurance				<.0001
Private	43.8	23.0	73.4	
Medicaid	43.4	61.7	17.4	
Self-pay	12.8	15.3	9.2	
Tobacco user	7.6	10.6	3.3	<.0001
Alcohol drinking	0.8	0.8	0.8	0.9617
Parity				<.0001
No previous live birth	44.7	43.1	46.9	
1 prior live birth	28.5	27.0	30.7	
2 live births	14.9	15.9	13.5	
≥3 live births	11.9	14.0	9.0	
Adequacy of prenatal care^b^				<.0001
Inadequate/intermediate	30.8	36.5	22.7	
Adequate	44.8	41.2	49.9	
Adequate plus	24.5	22.4	27.5	
Previous preterm birth	0.7	0.7	0.7	0.5951
Preterm birth	10.8	12.8	8.0	<.0001
*Neighborhood-level characteristics*				
Community violence^c^				<.0001
Quartile 1	34.7	24.9	48.6	
Quartile 2	17.9	20.0	15.0	
Quartile 3	30.1	33.2	25.7	
Quartile 4	17.3	21.8	10.8	
Female-headed household, % [mean (SD)]	41.9 (13.7)	46.3 (9.6)	35.6 (15.9)	<.0001^d^
Poverty, % [mean (SD)]	25.3 (9.5)	27.4 (7.7)	22.4 (10.8)	<.0001^d^
Black, % [mean (SD)]	56.5 (22.1)	63.8 (14.5)	46.1 (26.4)	<.0001^d^

^a^Chi square testing for differences between paternity statuses.

^b^Adequacy of prenatal care measured by Kotelchuck index.

^c^Quartiles 1–4 indicate lowest to highest community violence rates.

^d^ *t*-test for difference in mean between paternity statuses.

**Table 2:** Deviance tests for best model fit.

Model	−2LL	df	Δ−2LL	Δdf	*p* value
Model 0	22201.15	126	—	—	—
Model 1	22103.63	27388	97.52	27,262	<0.0001
Model 2	18267.89	27049	3835.74	339	<0.0001
Model 3^*∗∗*^	15038.42	26973	3229.47	76	<0.0001

^*∗∗*^Best fitting model;

Model 0: unconditional model;

Model 1: paternity status only;

Model 2: paternity status and individual-level characteristics;

Model 3: paternity status, individual-level, and neighborhood-level characteristics;

−2LL = −2Loglikelihood, Δ = change or difference;

Bonferroni correction, *p* < 0.0125 for statistical significance.

**Table 3:** Multilevel regression analysis showing racial/ethnic differences in the modifying effect of community violence on the association between paternity status and preterm birth.

Paternity status	Preterm birth adjusted odds ratio^**a**^ (model 3) (95% CI)
*Non-Hispanic White*	
Quartile 1	
No paternity established	1.42 (0.95–2.12)
Paternity established	Ref.
Quartile 2	
No paternity established	1.45 (0.57–3.71)
Paternity established	Ref.
Quartile 3	
No paternity established	3.12 (2.67–6.32)^*∗*^
Paternity established	Ref.
Quartile 4	
No paternity established	2.99 (1.17–7.66)^*∗*^
Paternity established	Ref.
*Non-Hispanic Black*	
Quartile 1	
No paternity established	1.16 (0.85–1.58)
Paternity established	Ref.
Quartile 2	
No paternity established	1.32 (0.82–2.12)
Paternity established	Ref.
Quartile 3	
No paternity established	1.64 (1.24–2.16)^*∗*^
Paternity established	Ref.
Quartile 4	
No paternity established	1.05 (0.74–1.49)
Paternity established	Ref.
*Hispanic*	
Quartile 1	
No paternity established	1.29 (0.65–2.55)
Paternity established	Ref.
Quartile 2	
No paternity established	1.34 (0.67–2.69)
Paternity established	Ref.
Quartile 3	
No paternity established	—^b^
Paternity established	
Quartile 4	
No paternity established	—^b^
Paternity established	

“Term birth” is reference category for preterm birth.

^a^Best fitted model (model 3) adjusted for maternal age, maternal education, insurance, tobacco use, alcohol drinking, adequacy of prenatal care, parity, previous preterm birth, percentage of non-Hispanic Black population, percentage of female-headed households, and percentage of individuals living <100% of the 2010 Federal Poverty Level.

^b^Unable to run regression analysis due to small numbers ^*∗*^*p* < 0.05.
